# How many AIDS epidemics can occur in São Paulo city?

**DOI:** 10.11606/S1518-8787.2018052000006

**Published:** 2018-05-18

**Authors:** Breno Souza de Aguiar, Cassia Maria Buchalla, Francisco Chiaravalloti

**Affiliations:** I Universidade de São Paulo. Faculdade de Saúde Pública. Programa de Pós-Graduação em Saúde Pública. São Paulo, SP, Brasil; II Universidade de São Paulo. Faculdade de Saúde Pública. Departamento de Epidemiologia. São Paulo, SP, Brasil

**Keywords:** Acquired Immunodeficiency Syndrome, epidemiology, Risk Factors, Socioeconomic Factors, Cluster Analysis, Spatial Analysis

## Abstract

**OBJECTIVE:**

An ecological study describring the spatial characteristics of AIDS in São Paulo city between 2001 and 2010 according to the place of residence of reported cases in adults.

**METHODS:**

The AIDS reported cases (28,146), grouped by sex, were geocodified (25,969) and linked with a census tract database (18,953). Case and population at risk data supplied spatial cluster identification and relative risk estimate by the scan method, using the discrete Poisson model. Incidence rate and proportional distribution allowed comparing people living in the high-risk clusters areas to other locations by age, race/ethnicity, schooling and transmission category.

**RESULTS:**

The AIDS incidence rate decreased in both sexes except among young men and older people. The identification of spatial high-risk clusters showed that the decrease of AIDS did not occur in the same way in the city. Clusters located in the central area presented the highest AIDS incidence rates (245.7/100,000 men), especially among black women (RR = 7.9), men who have sex with men (66.2%) and injection drug users (10.7%) participation. In peripheral clusters, identified only in the female population, the epidemic can be related to the poverty of these women (22.5% low education level). Residents in the north and central-south areas of the city are generally black, with little schooling, and predominantly heterosexually infected.

**CONCLUSIONS:**

The study of spatial clusters using a census tract helps to determine epidemiological patterns inside the city and in specific populations. Spatial stratification and key population epidemiological patterns were identified in four regions in São Paulo city.

## INTRODUCTION

The spread of HIV infection and AIDS reveals a multidimensional epidemic that has shown important epidemiological changes^1^, and that results from social conditions, economic, and cultural differences^2^ ^,^ ^3^. The study of spatial AIDS epidemiology is complex^4^ due to the different contexts of the disease – individual and collective, wider coverage and complexity.

Bastos and Barcelos^5^ evaluated the spatial distribution of AIDS cases in Brazil between 1987 and 1993 and indicated the attractive force of São Paulo state as the epidemic diffusion region in the country, not only for its population density but for concentrating exchange activities and social interaction^6^ with the rest of the country. In 1994, Grangeiro^7^ evaluated AIDS cases in 1983–1984 in São Paulo city by transmission category and identified five homogeneous regions according to their pattern of transmission.

Farias and Cardoso^8^ reported trends in AIDS morbidity and mortality from 1994 to 2002 with the socioeconomic conditions in São Paulo city using the “*S*ocial Exclusion and Inclusion City Map”^9^. The authors observed a decrease in incidence rates during the study period, with less intensity in the peripheral areas of the city – where social exclusion is greater.

In a study that evaluated magnitude and AIDS trend in Brazilian cities between 2002 and 2006^10^, the authors set the epidemic in São Paulo as of great magnitude, stable and with epidemiological features that have been consolidated since 1990.

Spatial analysis methods in Public Health have been used in ecological studies^11^ to incorporate variables that reflect the dynamics of collective events to health data geoprocessing. Among these methods, the scan statistic allows the identification of spatial clusters from minimum territorial aggregations^12^.

The rationale for studying AIDS spatial patterns in São Paulo city is related to the fact that it is a large, highly urbanized and heterogeneous city with an intense social exchange that contributes to the maintenance of the AIDS epidemic. The city is an AIDS diffusion hub for other municipalities in a mixed pattern of diffusion (contiguity and hierarchical)^6^.

In addition, only a few authors have analyzed the AIDS epidemic in sub-municipal scale^13^, with most studies in the central area or certain groups, generally using Administrative District as an analysis unit. In São Paulo city, the inequalities associated with AIDS cases can be expressed in different spatial patterns. This study aimed to describe these patterns in São Paulo city between 2001 and 2010, based on the analysis of adult cases according to place of residence.

## METHODS

**Figure 1:**
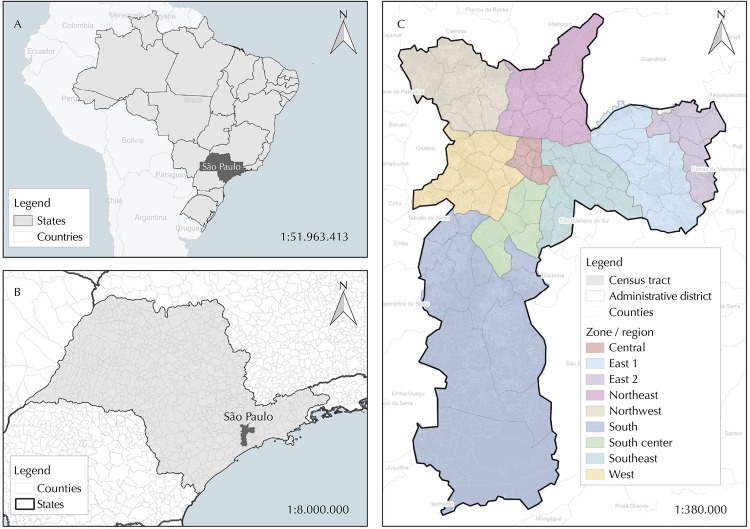
Brazil (A), São Paulo state (B) and São Paulo city by Zone/Region (C).

Source: IBGE, 2010; SEMPLA/São Paulo city, 2012.

São Paulo city, located in São Paulo state at the Southeast region, is divided into 18,953 census tracts, 96 Administrative Districts and nine regions or areas taking into account the geographical position and historic occupation of the city (Figure 1). With a high degree of urbanization and a Municipal Human Development Index of 0.79, this city, compounded with 38 other counties, has the higher density population in Brazil and the fourth in the world^14^.

This descriptive and ecological study refers to AIDS reported cases in the population older than 13 years old in São Paulo city, with diagnosis dated between January/2001 and December/2010. The AIDS cases were obtained from the Notifiable Disease Information System (SINAN W/NET), Disease Control Center/Health Surveillance Coordination (CCD/COVISA SMS-SP) in July/2011, analyzed by sex, age group, race/ethnicity, schooling, transmission category, and geocoded by place of residence. Cases were geocoded by metric interpolation in the TerraView 4.1.0 software using the address layer (Lat/Long, SAD69) provided by São Paulo City Health Department.

The scan method analysis^12^ was done by sex using the discrete Poisson model – which uses the population at risk as the denominator – and standardized by age^10^. To identify spatial clusters, three databases were incorporated in the Sat Scan 9.1.1 software: the first linked cases by age group to census tract; the second contained the population in census tracts by age group, and the third had centroids geographical coordinates of census tracts. These populations were characterized using the Demographic Information Database: Results of Census Sector Universe^15^ as a reference.

Whereas clusters identification depends on the methodology and parameters defined^16^, such as cluster format, spatial overlap, maximum cluster size and replication number^12^, the following conditions were considered to identify spatial clusters: circular; nonoccurrence of geographical overlap; maximum size equal to 50% of the exposed population (to minimize selection bias)^12^; and the statistical significance was tested by the Monte Carlo method using 999 interactions with p < 0.05.

Clusters were printed in census tracts and Administrative Districts^17^ maps of São Paulo city. Cluster information, such as census tracts, cases, relative risks (RR) and related zone or region, were presented.

For greater magnitude and severity clusters, we estimated the annual incidence rates in 2001, 2010 and in the entire 2001/2010 period, age-adjusted; age-specific and color-specific incidence rates were also estimated. To estimate the annual incidence rate, cases by year of diagnosis were divided by the population, calculated from annual geometric growth rate 2000–2010 by sex and age group on cluster corresponding area.

Regarding the schooling and transmission categories, proportional distribution, including chi-squared statistics, was used to compare clusters. Education and sexual behavior in intra municipal scale data were available for weighting areas^15^ and Health Department Administrative Regions^18^, respectively, preventing the rate calculation. Incidence rate and proportional distribution cluster data were compared to the São Paulo city population by sex.

The study was approved by the Ethics Committees of Faculdade de Saúde Pública of the Universidade de São Paulo (CAAE 0132.0.207.162-11; Office COEP/016/12) and São Paulo City Health Department (Process 24/12 CEP/SMS).

## RESULTS

A total of 28,146 cases were reported in adults living in São Paulo city (18,709 males and 9,437 females) between 2001 and 2010. Geocoding process has excluded 2,177 cases – 455 (1.6%) had no local data; 1,425 (5.1%) had incomplete address, wrong data or were not found in address layer; 38 (0.1%) showed topological problems between the layers (i.e., were found outside the city limits); and 259 (0.9%) were associated with census tracts with cases higher than the population. Geocoding rate was about 92.3% (17,298 male and 8,671 female cases).

There was a decrease in AIDS incidence rate, age-adjusted, for both sexes, except for males between 13 to 29 years old and in the older population (60 or over). In males, the AIDS incidence rate decreased from 62.1 cases per 100,000 men in 2001 to 34.5 in 2010. In females, these values corresponded to 24.5 and 11.6, respectively. The sex ratio, 2.5 AIDS cases in males for each case in females since 2001, showed a slight increase in 2007 and reached 3.0 in 2010. The incidence rate was higher for people aged 30 to 59 years old than in other age groups in both sexes.

**Figure 2:**
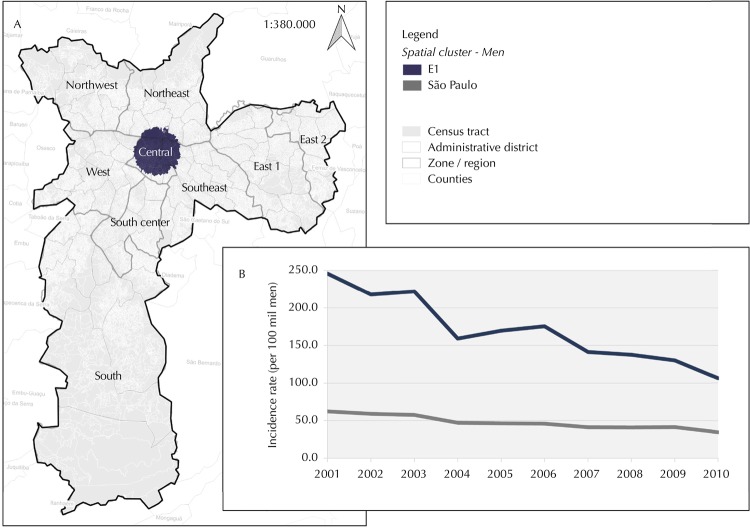
AIDS cases cluster (A) and annual incidence rate, per 100,000 men, age-adjusted (B). São Paulo city, 2001–2010.

Source: Administrative district – Sao Paulo city, 1996. Census tract and counties – IBGE, 2010. Spatial cluster – SINAN, July/2011. Zone/region – SEMPLA/São Paulo city, 2012.

**Figure 3:**
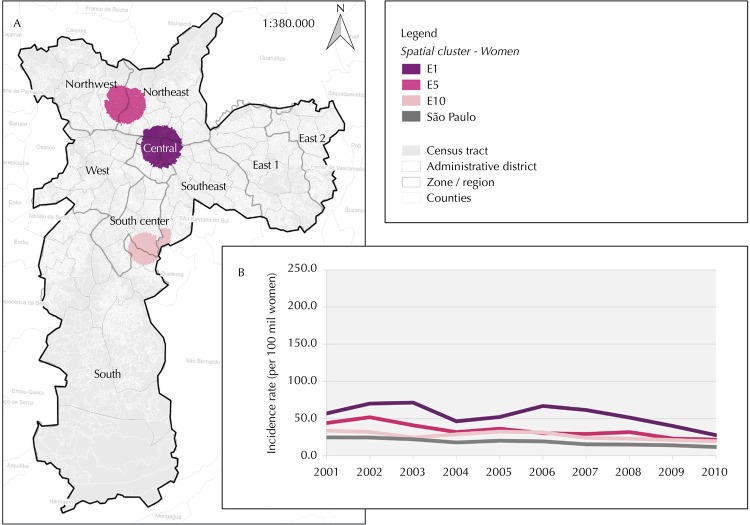
AIDS cases clusters (A) and annual incidence rate, per 100,000 women, age-adjusted (B). São Paulo city, 2001–2010.

Source: Administrative district – Sao Paulo city, 1996. Census tract and counties – IBGE, 2010. Spatial cluster – SINAN, July/2011. Zone/region – SEMPLA/São Paulo city, 2012.

The AIDS reported cases clusters, three in male population and 10 in female, were identified in São Paulo city between 2001 and 2010. Considering magnitude and severity, four clusters were highlighted, one in the male population: E1 (Figure 2) and three in the female population: E4, E5 and E10 (Figure 3).

The E1 cluster, comprising 4.4% all of census tracts, presented 2,684 (15.5%) cases in males during the studied year interval, with relative risk of 4.3 compared to male residents outside the cluster. Cluster identification, such as census tracts, cases, relative risk information and related zone or region are presented in Table 1. Table 2 shows the AIDS incidence rates, age-adjusted, in 2001, 2010 and in the period, age- and race-specific, as well as the proportional distributions by schooling and transmission category of the clusters.

**Table 1:** Cluster identification, census tract, cases, relative risk (RR), and related zone/region by sex. São Paulo city, 2001–2010.

Id	Census track		Cases		RR	Zone/Region
	
	n	%	n	%		
Spatial cluster – Male						
E1	829	4.4	2,684	15.5	4.3	Central, Southeast and Northeast
Spatial cluster – Female						
E4	429	2.3	506	5.8	3.0	Central, Southeast and Northeast
E5	754	4.0	712	8.2	1.9	Northwest and Northeast
E10	503	2.7	346	4.0	1.5	South, South Center and Southeast

Source: SINAN CCD/COVISA – SMS/SP; IBGE 2010.

**Table 2:** Clusters incidence rate in 2001, 2010 and entire interval (age-adjusted), age-specific and race-specific (per 100,000 inhabitants), schooling and transmission category proportional distribution (per 100 cases) by sex. São Paulo city, 2001–2010.

Variable	Male		Female			
	
	E1	MSP	E4	E5	E10	FSP
Incidence rate (per 100,000 inhabitants)						
2001	245.7	62.1	56.8	43.9	33.9	24.5
2010	106.3	34.5	27.6	21.7	19.4	11.6
Interval	170.4	47.6	54.5	34.0	27.1	18.8
Age-specific (age group)						
13–29	101.3	23.6	40.6	21.6	16.4	14.0
30–59	251.0	65.8	77.7	51.1	40.4	27.4
≥ 60	45.3	11.3	7.4	6.7	6.4	3.9
Race-specific^a^						
White	102.0	34.9	28.1	19.7	16.3	13.8
Black	342.8	57.0	221.4	67.1	45.1	31.5
Brown	192.6	27.6	99.3	27.3	30.4	14.9
Proportional distribution (per 100 cases)						
Schooling (years)^b^						
None/1–3	10.2	12.8	16.7^c^	22.5	22.5^c^	18.5
4–7	21.4	29.3	38.6	39.2	40.7	37.2
8–11	40.2	39.9	35.3	33.4	31.6	37.0
12 or more	28.2	18.0	9.3	4.9	5.2	7.3
Transmission category^d^						
Homosexual/Bisexual	66.2	46.0	0.3	0.5^c^	0.3^c^	0.3
Heterosexual	24.9	42.2	88.7	95.1	94.9	94.0
Injection drug user	8.7	11.3	10.7	3.7	4.1	5.2
Others^e^	0.2	0.5	0.3	0.7	0.7	0.6

Source: SINAN CCD/COVISA – SMS/SP, 2011; IBGE, 2010.

E1: male spatial cluster in Central, Southeast and Northeast; MSP: male population in São Paulo city; E4: female spatial cluster in Central, Southeast and Northeast; E5: female spatial cluster in Northwest and Northeast; E10: female spatial cluster in South, South Center and Southeast; FSP: female population in São Paulo city

^a^ “Unknown information” on race/ethnicity variable E1 25.3%, MSP 22.30%, E4 22.1%, E5 24.7%, E10 19.0% and FSP 21.3%.

^b^ “Unknown information” on schooling variable E1 23.4%, MSP 20.4%, E4 27.9%, E5 20.1%, E10 17.6% and FSP 18.8%.

^c^ Results not statistically significant (p > 0.05).

^d^ “Unknown information” on transmission category variable E1 23.0%, MSP 24.1%, E4 28.3%, E5 16.3%, E10 13.7% and FSP 19.5%.

^e^ Mother-to-child transmission, accident with biological material, hemophiliac.

Central clusters, in both sexes, showed different characteristics of peripheral clusters: higher incidence rates and relative risks, including among young people; greater inequality between whites and blacks; and variability in transmission category. The E1 cluster showed a decrease of 56.7% in incidence rate during this time interval, while in the male population this figure amounted to 44.4%. All female clusters showed lower decrease in incidence rate compared to São Paulo’s female population, which was 52.7%.

Race/ethnicity data quality has improved during this time interval, especially between 2002 and 2003. In 2001, it was 74.7% AIDS reported cases had no information; in 2010, it was 5.1%. Considering the data system improvement since 2003, there has been an increase in non-white cases; however, white men represent the majority of cases during the study year period. In the historical analysis (2001–2010), the number of AIDS cases in white women was always higher than for black or brown women, except for 2010, when the number of cases for black/brown women was higher.

Race-specific AIDS incidence rates in central clusters were higher than in peripheral clusters. The black-specific incidence rate for the E1 cluster was 2.4 times higher than the white one; this amount correspond to 0.6 in the city’s male population. The E4 cluster excelled, presenting black-specific incidence rate 6.9 times higher than in white women and higher than that observed for the entire study area. Peripheral clusters E5 and E10 exhibited black-specific incidence rates respectively 1.8 and 2.4 times higher. In the São Paulo female population, this amount represents a slightly lower value, 1.3.

Among the cases analyzed, 22.1% and 14.7% had no schooling data information in 2001 and in 2010, respectively. Although the lack of this information rate has dropped, there was an increase in schooling among the reported cases in the interval assessed for both sexes. The E1 cluster showed that 28.2% of cases had 12 years or more of schooling. However, there was a higher concentration of less educated female cases – only 7.3% reported 12 years or more of schooling. The E4 cluster has proportionally higher education compared to the female population overall; though this difference was not statistically significant. Peripheral clusters E5 and E10 showed a higher proportion of less educated female cases.

The lack of information on transmission category decreased between 2001 and 2010. In the male population, it decreased from 30.8% in 2001 to 16.5% in 2010, while in the female population, it went from 24.4% to 13.4%. Regarding sexual transmission, relative cases increased in men who have sex with men (MSM) and have remained stable in heterosexual ones between 2001 and 2010. Blood transmission type – injecting drug use (IDU), hemophiliac, an accident with biological material and vertical transmission accounted for 14.6% of total cases in adults in 2001 and lost expression going to 6.3% in 2010.

The E1 cluster presented 66.2% cases in MSM, while in the city’s male population this figure amounted to 46.0%. Regarding women, the proportion of IDU cases was higher in the E4 cluster, located in the central region, compared to peripheral clusters E5 and E10.

## DISCUSSION

The SINAN W/NET AIDS quality data related to place residence in São Paulo city between 2001 and 2010 was quite adequate – 98.4% of the cases had complete information. This high proportion can suggest good efficacy of AIDS surveillance, as this proportion on the national AIDS database was 94.8%^19^. Correct addresses, including standardized street name, number and ZIP code, is used in the geocoding step, so the proper completion of Notification or Investigation Sheet is a key element for consistent spatial analysis. In this study, the geocoding rate was 92.3%.

The SINAN is the most important system for AIDS survailance^20^. However, some limitations, such as poor linkage with other database system and data surveillance focused in general population, in spite of key-population^21^, are associated with SINAN data analysis.

The AIDS incidence rate decreased in São Paulo city between 2001 and 2010, as well as described by Grangeiro et al.^10^ in 2010 for other highly urbanized federal capitals, with largest sex ratio, diversified transmission category and older epidemics. Some conditions played an important role in this decrease, such as expanding access to early diagnosis of HIV infection^22^ and the beginning of highly active antiretroviral therapy (HAART) in due time^23^, which helped to improve the life quality of people living with HIV (PLHIV). However, the occurrence of spatial clusters evidenced that this decrease did not occur homogeneously within the city, from the point of view of the most vulnerable populations^24^ ^,^ ^25^ or regions or geographical areas, as described by Szwarcwald et al.^26^ for other regions.

Municipal aggregate unit is not enough to analyze AIDS epidemic in large urban centers like São Paulo since it can homogenize completely distinct lifestyles and health conditions. Epidemic heterogeneity in São Paulo city can be analyzed according to Administrative Districts or weighting areas. However, the use of census tract as analysis unit enables us to better discriminate the territory, as it avoids the artificial partitioning of the territory, based on political-administrative criteria that cannot be related with AIDS, revealing health inequities^27^.

Central and peripheral clusters showed different characteristics between them and in relation to São Paulo city by sex regarding the magnitude and severity^28^. Central clusters differ from peripherals because they have higher AIDS incidence rates, greater social inequalities (expressed by race/ethnicity and schooling variables) and greater variety in relation to transmission category – higher proportion of MSM and IDU^27^. In the central region, more urbanized, with intense exchange and social interaction, the epidemic has higher incidence rates and is concentrated in key populations, as observed in the Knowledge, Behavior and Practices Survey^18^ (PECAP-MSP). In the peripheral clusters, identified only in the female population, the epidemic may be related to poverty classic indexes^8^, with a predominance of heterosexual transmission in black, less-educated women.

Other spatial clusters, two identified in male and seven in the female population (data not presented), were composed of few census tracts (less than 0.1%) and cases (less than 0.2%). In these cases, high relative risk values could be related to the variability associated with observations and not to AIDS illness risk^29^.

No clusters were identified in the eastern, western and southern areas of the city. In this case, we must consider that spatial analysis was restricted to São Paulo city, without considering the municipality’s surroundings. Spatial analysis data in the São Paulo metropolitan region could modify the results of the study, especially in peripheral regions.

In São Paulo, as in other large cities, MSM prevails as the population most affected in all periods. The PECAP-MSP^18^ pointed out that 8.6% of men surveyed have had sex with another man in life; however, the proportion of cases among MSM accounted for half of the cases in the city and was more than 65% in the E1 cluster. The AIDS epidemiological pattern has not changed in central region in the last three decades, consolidating its position as an old epidemic^3^, similar to the 80’s – when it was characterized as a region homogeneous A^7^, with a predominance of cases among MSM^30^.

Grangeiro et al.^27^, in a study done in Brazil between 2002 and 2006, assessed temporal patterns in relation to the transmission category and showed that the beginning of the epidemic occurred in more educated homosexual men and was later spread to the poorest populations. The mobility of MSM from peripheral regions or other counties to the central region of São Paulo city seems to be what keeps the standard of the illness in the region. In this case, the variable time was more important to consolidate the epidemiological pattern than to change it significantly^10^. In other words, it can be assumed that the population of MSM in the central region is not likely limited, because it is always renewed^a^. However, PECAP-MSP^18^ pointed out that there is no difference in the proportion of MSM by Health Department Administrative Regions in São Paulo city.

Geographical overlap of spatial clusters E1 to E4 suggests a close relationship on AIDS illness pattern in both sexes at Central region. The AIDS incidence rate in the E4 cluster was higher than in the male population. Research by Farias and Cardoso^8^ between 1994 and 2001 in São Paulo, noted a decreased sex ratio in all areas studied, except in the Central region, which had the highest sex ratio compared to peripheral regions. This shows the differences in the AIDS sexual transmission pattern in these territories.

In relation to female AIDS epidemic, the analysis of race/ethnicity and schooling variables in the E4 cluster suggests cohabitation of women with different sociodemographic profiles. In this case, the pauperization of the epidemic cannot be related to the classic indexes, but to social differences and characteristic of poverty pocket in urban centers^27^. According to São Paulo Civil Department (HABISP-SP), of 13,351 inhabitants living in slums in 2014, 42.0% lived in the E4 cluster area (data not shown). These data may indicate, in addition to intense social interaction typical of large urban centers, huge social inequalities that become evident in São Paulo city’s central area. The identification of spatial clusters in females, especially in peripheral northeast and southeast regions, may be associated with their worse living conditions^21^ ^,^ ^31^.

Black to white race-specific AIDS incidence rate rose in São Paulo city^25^, especially for the female population in the central region. The PECAP-MSP^18^ found that blacks have less awareness about the transmission and prevention of HIV infection than whites, and black women have less awareness of female condoms. Also, blacks showed lack of awareness of public health services that perform HIV test and made a less anti-HIV tests in life compared to whites. Study by Khoury et al.^23^ in São Paulo city showed that more than half of people living with HIV surveyed sought treatment after already being diagnosed with AIDS, and this number was higher among blacks. It is possible that less access to health services in São Paulo city, including late diagnosis and HAART at inappropriate time, contributes to the increase in AIDS incidence rates for black individuals.

When evaluating the race/ethnicity, schooling, and transmission category variables to understand AIDS morbidity spatial pattern, we must consider the high proportion of unreported data. The AIDS Municipal Program and CCD/COVISA have worked on improving race/ethnicity quality data observed since 2003^32^. Regarding education, it is emphasized that the data were collected into two versions of SINAN: W (up to 2006) and NET (2001/2010). The W version considered the number of schooling with approval, while the NET used grade completed. Information related to the transmission category improved during this period; however, it is necessary to evaluate the high proportion of ignored cases, especially in the central area.

Despite limited analysis for poor completeness in some variables, one might think that the complexity of the central area (e.g., high urbanization) makes inequalities so evident as to distinguish populations in relation to AIDS pattern morbidity. Health promotion and AIDS prevention programs must prioritize the most vulnerable populations – young MSM and people living in poverty in the central region as to promote equity in health. Other types of studies are necessary to assess the quality of health care for PLHIV and thus understand the inequalities between different population groups inside the county.

Combating stigma and prejudice against sexual behavior, race/ethnicity or HIV status, increasing the supply of prevention inputs, early diagnosis of HIV infection and HAART initiation in due time are fundamental in AIDS epidemic control in São Paulo city. The identification of high-risk clusters and these epidemiological patterns characterization enable the understanding of health needs in these populations, which are priority regions for the development of health promotion actions, as well as AIDS prevention and control in São Paulo city.

In the central region, the actions should be focused on specific interventions, such as pre-exposure and post-exposure prophylaxis, in key populations (MSM, sex workers and people with abusive use of psychoactive substances). While in peripheral regions, the prevention and control of disease should include, in addition to key populations, the general public, in line with social care and education services.

## CONCLUSIONS

The AIDS cases spatial analysis, using scan method and census tract, identified high-risk clusters in São Paulo city from 2001 to 2010 and key population epidemiological patterns.

Clusters located in the central area, Central region and part of the Southeast and North, presented the highest AIDS incidence rates, especially in black individuals, MSM and IDU participation.

In peripheral clusters, identified only in female population, the epidemic can be related to the poverty of these women. Residents in the North and Central-South of the city are generally black, have little schooling and are predominantly heterosexually infected.

## Footnotes

a Gupta S, Anderson RM, May RM. Networks of sexual contacts: implications for the pattern of spread of HIV. *AIDS*. 1989;3(12):807-17. https://doi.org/10.1097/00002030-198912000-00005
